# INSIDE PAHPA: A GSA POLICY INTERN’S JOURNEY TO THE INTERSECTION OF POLICY AND PREPAREDNESS

**DOI:** 10.1093/geroni/igad104.1236

**Published:** 2023-12-21

**Authors:** Alisha Thompson, Bailee Brekke, Christina Mu, Patricia D’Antonio

**Affiliations:** LSU, Baton Rouge, Louisiana, United States; Miami University, Oxford, Ohio, United States; University of South Florida, Tampa, Florida, United States; The Gerontological Society of America, Washington, District of Columbia, United States

## Abstract

Public health and safety are facing increasingly severe threats from natural disasters, infectious diseases, and emergencies caused by human actions, both intentional and unintentional. These incidents now impact a larger number of people and cover broader geographical areas compared to the past. Chief among those most affected are older people, as highlighted during the COVID-19 pandemic and other crises. Addressing these disparities is crucial, and the federal government plays a pivotal role in tackling this issue. Much of my research up to this point has focused on preparedness among older people for emergencies that threaten public health. My experience as a GSA Summer Policy Intern has significantly influenced my understanding of the relationship between public health emergencies and aging policy. Through witnessing the legislative process at the national level, monitoring resource allocation in the federal budget, and attending Congressional hearings, I gained valuable insights into the complexities of this field. My perspective was further shaped by closely monitoring, synthesizing, and disseminating information about the 2023 Reauthorization of the Pandemic and All-Hazards Preparedness Act (PAHPA). Additionally, I provided valuable feedback on the relevance of this bill to the interests of GSA and contributed to comments on a bipartisan discussion draft related to this legislation. Equipped with this newfound insight and understanding, I am confident that I will be a more effective advocate and scholar in the field of aging policy.

